# The influence of price value on purchase intention among patients with chronic diseases in medical e-commerce during the COVID-19 pandemic in China

**DOI:** 10.3389/fpubh.2023.1081196

**Published:** 2023-02-01

**Authors:** Linlin Han, Xu Han

**Affiliations:** ^1^Faculty of Business Information, Shanghai Business School, Shanghai, China; ^2^School of Information Engineering, Minzu University of China, Beijing, China

**Keywords:** purchase intention, medical e-commerce, theory of planned behavior, price value, patients with chronic diseases, COVID-19 pandemic

## Abstract

**Background:**

During the COVID-19 pandemic, medical e-commerce (MEC) has provided a way for patients with chronic diseases to purchase drugs online to maintain social distancing, decrease the risk of infection and community transmission, and relieve the burden on medical resources. Therefore, research which seeks to elucidate the drivers of purchase intention (PI) among patients with chronic diseases in MEC is vital. This study extended the theory of planned behavior (TPB) by integrating the price value (PV) variable into the original TPB framework and explored the effect of PV on patients' PI in MEC during the coronavirus pandemic.

**Methods:**

Empirical data was gathered from 414 Chinese participants. Structural equation modeling was applied to explore the mechanism of chronic patients' PI in MEC. In addition, this study also estimated the moderating effect of gender, income, and region and the mediating role of attitude (ATT), subjective norm (SN), and perceived behavioral control (PBC) between PV and PI.

**Results:**

Patients' PI in MEC is significantly affected by ATT, SN, and PBC. PV delivers significant influence on ATT, SN, PBC, and PI, with PV having the strongest effect on ATT. Gender, income, and region can significantly moderate the relationship between PV and ATT.

**Conclusion:**

These findings can contribute to design targeted interventions to increase the adoption of MEC for patients with chronic diseases, decrease infection rates, and alleviate the strain on medical resources in the COVID-19 era.

## 1. Introduction

COVID-19 (coronavirus disease), a highly contagious pneumonia, has become a pandemic (1). Several studies have suggested that the transmission of the 2019 novel coronavirus infection occurs primarily through contact transmission and respiratory droplets (2, 3). In China, people were requested to stay at home and practice social distancing during the COVID-19 outbreak to overcome its rapid spread (4). During this time, many patients, especially people with chronic diseases, had difficulty accessing medical aid, as valuable resources in hospitals were allocated toward mitigating COVID-19 (5). In addition, gathering in hospitals increases the risk of nosocomial infection for patients who already have various diseases or have low immunity; it also significantly increases the risk of community transmission (6). E-commerce platforms, especially medical e-commerce (MEC) platforms, naturally have such characteristics to avoid close social contact (7, 8). Therefore, from the perspective of decreasing the risk of face-to-face contact in hospitals, purchasing drugs from MEC platforms, which makes the online search for drugs more accessible to patients than on-site, is helpful for people with medical demands during the COVID-19 pandemic (9). Most MEC platforms provide excellent customer service, quality assurance, and rapid delivery, making purchasing drugs online stress-free and convenient (10). After several years of development in China, the industrial structure of MEC, which ensures medication safety through strict laws and regulations, has developed rapidly. Take JD Health, a renowned MEC platform, as an example, many patients with chronic diseases purchased drugs online during the outbreak of COVID-19 in 2020. As of December 31, 2021, JD Health's annual active user number reached 123 million, representing a net increase of 33.56 million active users compared to 2020. During the reporting period in 2021, JD Health's total revenue was 30.68 billion RMB, up 58.3% year-on-year. The total trading volume of MEC was ~195 billion RMB in 2018, which reached 255.4 billion RMB in 2019, representing a growth rate of 31%, thereby indicating a strong growth trend of the MEC industry, with substantial room for development (11).

The Report of Nutrition and Chronic Diseases on Chinese Residents published by the National Health Commission in 2020 shows that more than 300 million people accounted for 86.6% of all deaths owing to chronic diseases, and patients with chronic disease accounted for 70% of the overall disease burden in the country. In 2020, the mortality rate of patients with comorbidities was 1.4%. It is significantly higher than that without comorbidities (12). In this context, strengthening the protection of patients with chronic diseases can significantly reduce mortality. Along with the policy released by China National Health Commission issued during the coronavirus pandemic, the government encouraged designated medical institutions to provide “do not meet” drug purchase services and realized online medical insurance settlement for qualified online medical services (13). Consequently, MEC platforms would improve the effectiveness of doctors' work, which would significantly optimize medical resource allocation efficiency.

However, research on patients' purchase intention (PI) among patients with chronic diseases in MEC is minimal. Existing studies have explored digital healthcare retail (10, 14–17). For example, Yang et al. (10) revealed that patients' experience was exclusive to internet pharmacy services, and patient compliance can be enhanced by their experience and network social support. Liu et al. (14) proposed an algorithm and used users' purchase data to develop users' diseases to provide product recommendations for pharmaceutical e-commerce platforms. Sreejesh et al. (15) examined how the technology-enabled service co-creation affected patients' service purchase behavior in medical and health services retail. Zehnder et al. (16) studied the growth of Swiss community pharmacies on the internet over the period 2000–2003, which showed that pharmacy-group portals were promoters of internet pharmacies. Ma (17) investigated the factors underlying non-adopters' intention to purchase drugs online *via* the technology acceptance model. Few studies have considered the factors that stimulate patients, especially patients with chronic diseases, to form PI on MEC platforms.

Previous researchers have used the theory of planned behavior (TPB) from multiple disciplines to predict various behaviors [e.g., (18–21)]. The TPB model is also adopted to explore people's health behavioral intentions (22, 23). Although the TPB model provides a conceptual framework for human social behavior research, Ajzen (24) suggested that researchers could extend the TPB model to explain or predict complicated psychological mechanisms. Many previous studies have added factors to extend the TPB model and improve its effectiveness and applicability. Price value (PV) is an important factor that could influence patients' PI in MEC (25–28). For example, Crawford (25) stated that easy for price comparisons were an important advantage of online pharmacies. Brown et al. (26) administered a cross-sectional anonymous survey and found that the population of online pharmacies was highly associated with the costs of prescription drugs while searching for opportunities for cost savings increased consumers' online PI. Fittler et al. (27) found that patients may care about discounts, bonuses, and gifts from online pharmacies, which also affected patients' attitudes toward purchasing drugs online. Liu et al. (28) analyzed consumers' online pharmacy purchasing comments and found that price (including affordable and expensive) was an important attribute, which affected patients' satisfaction. Chronic patients need to purchase drugs continuously and are more sensitive to prices (29). Accordingly, we add PV to help explain and predict the PI in MEC among patients with chronic diseases during the coronavirus pandemic.

Therefore, this paper aimed to (a) investigate the mechanism of PV on chronic patients' PI in MEC during the coronavirus pandemic using an extended TPB model, and (b) examine the moderating effects of the relationships within the model, subgroups classified by gender, income, and region. The findings of this study can provide new insights for governments, business practitioners, and researchers to promote the adoption of online drug purchasing by patients with chronic diseases, decrease infection rates, and improve the utilization efficiency of medical resources during the COVID-19 pandemic.

## 2. Theoretical background and hypotheses

### 2.1. Theoretical background

The present study used the TPB as a framework to employ the psychological mechanism of chronic patients' PI in MEC during the coronavirus pandemic. The TPB model has four primary constructs, including behavioral intention (BI), attitude (ATT), subjective norm (SN), and perceived behavioral control (PBC). Notably, ATT, SN, and PBC are independent determinants of BI (30). The more favorable of the ATT, SN and PBC, the stronger the people's BI (31). Moreover, TPB is a validated model that has been used in many public health-related behavior explanations such as the intention to get coronavirus vaccines (32) and use traditional Chinese medicine (TCM) (33). Chang et al. (29) explored the factors that affect chronic patients' PI in offline pharmacies based on the TPB model. In the TPB model, the main assumption is that people are rational in their decision-making, such that the actual behavior can be predicted *via* cognitive approaches (24). In this study, the patients' PI in MEC depends on several predictors, including ATT toward purchasing drugs online, SN for purchasing drugs from MEC platforms, and PBC over MEC platforms.

### 2.2. Research hypotheses

#### 2.2.1. Attitude

Notably, ATT is defined as an individual's positive or negative evaluation of a particular behavior (24). According to the TPB concept, PI can be affected by one's ATT toward the behavior. When appraising the medical behaviors during the COVID-19 epidemic, people are likely to evaluate various aspects of medical requests, such as the ATT toward the level of risk they are willing to take (34). In the present study, ATT means patients' overall estimation of MEC participation during the COVID-19 pandemic. When patients' ATT in MEC is positive, their PI in MEC is more likely to be positive. As such, this study proposes the following hypothesis:

H1: Attitude has a positive and direct influence on PI in MEC during the COVID-19 pandemic.

#### 2.2.2. Subjective norm

Moreover, SN is defined as individuals' perception that most people who have significant influence on them believe that they shall or shall not execute the behavior in question (24). Individuals may evaluate their beliefs and changes their consumption patterns, as induced by people have an important influence on them (35). Chang et al. (29) found that SN has a weak overall impact on offline pharmacy PI, as patients with chronic diseases have a relatively rich experience in drug use and purchase. However, the intention of patients with chronic diseases to purchase drugs on MEC platforms may be more susceptible to others because of the need to maintain social distancing during the coronavirus pandemic. If patients consider that people who can influence their behavior believe purchasing drugs from MEC platforms is a good choice during the COVID-19 pandemic, their PI in MEC will be enhanced. Hence, we propose the following hypothesis:

H2: Subjective norm has a positive and direct influence on PI in MEC during the COVID-19 pandemic.

#### 2.2.3. Perceived behavioral control

Furthermore, PBC refers to individuals' perceived ease or difficulty in performing a behavior (24). Some scholars state that PBC refers to people's perceived ability to control behavior (36). In the present study, PBC means patients have the resources, knowledge, and capacity to purchase drugs from the MEC platform during the COVID-19 pandemic. Strong PBC may induce positive PI. Based on the above-mentioned, this study proposes the following hypothesis:

H3: Perceived behavioral control has a positive and direct influence on PI in MEC during the COVID-19 pandemic.

#### 2.2.4. Price value

Finally, PV presents an overall evaluation of a product's utility and benefits, such as convenience, price, and time cost (37). A product or service's PV is generally determined by its monetary cost or price in conjunction with the quality of the offering (38). When the benefits are perceived to be larger than the price, PV will positively affect PI (39). Therefore, it is necessary to analyze PV's impact on chronic patients' intention to purchase drugs online. This study defines PV as the cognitive tradeoff patients make between the perceived benefits and monetary costs in the transaction online. Thus, we hypotheses discussed below:

H4a: PV has a positive and direct influence on ATT in MEC during the COVID-19 pandemic.
H4b: PV has a positive and direct influence on SN in MEC during the COVID-19 pandemic.
H4c: PV has a positive and direct influence on PBC in MEC during the COVID-19 pandemic.
H4d: PV has a positive and direct influence on PI in MEC during the COVID-19 pandemic.

**Figure 1:**
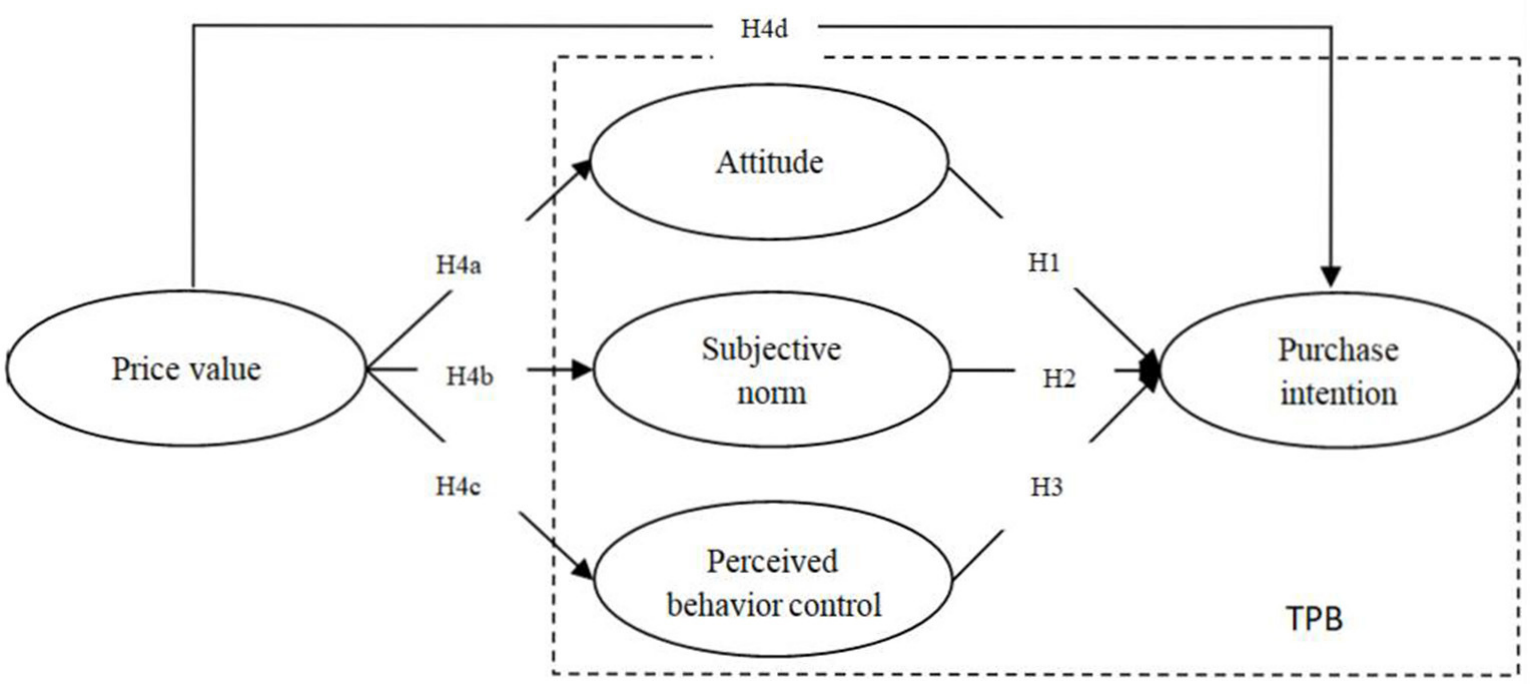
Conceptual model and hypotheses.

In summary, Figure 1 shows the framework of this research, and depicts the relationships between PV, ATT, SN, PBC, and PI in MEC during the COVID-19 pandemic.

## 3. Methodology

### 3.1. Survey design and measurement items

We performed an anonymous, cross-sectional survey in China. The questionnaire included three parts. The first part explained the objective of the survey and the definition of MEC platforms. The second part of the questionnaire contained the participants' demographic data. The third part contained 17 items (Table 1) measuring five constructs: ATT, SN, PBC, PV, and PI.

**Table 1:** Measurement instruments.

**Construct**	**Measurement items**	**References**
Attitude (ATT)	Please fill out the below questions about your attitude in MEC during the coronavirus pandemic.	Taylor et al. (40 )
	ATT1. Purchasing drugs from MEC platforms is a good idea.	
	ATT2. Purchasing drugs from MEC platforms is a wise idea.	
	ATT3. I like the idea of purchasing drugs from MEC platforms.	
	ATT4. Purchasing drugs from MEC platforms is a pleasant experience.	
Subjective norm (SN)	Please fill out the below questions about subjective norm in MEC during the coronavirus pandemic.	Venkatesh et al. (41)
	SN1. People who influence my behavior would think that I should purchase drugs from MEC platforms.	Mathieson (42)
	SN2. People who are important to me think that I should purchase drugs from MEC platforms.	
	SN3. People whose opinions I value prefer me to purchase drugs from MEC platforms.	
Perceived behavior control (PBC)	Please fill out the below questions about perceived behavior control in MEC during the coronavirus pandemic.	Taylor et al. (40)
	PBC1. I am able to purchase drugs from MEC platforms.	
	PBC2. Purchasing drugs from MEC platforms is entirely within my control.	
	PBC3. I have the resources, knowledge, and ability to purchase drugs from MEC platforms.	
Price value (PV)	Please fill out the below questions about price value in MEC during the coronavirus pandemic.	Venkatesh et al. (37)
	PV1. Drugs in MEC platforms are reasonably priced.	
	PV2. Drugs in MEC platforms are good value for money.	
	PV3. At the current price, MEC platforms provide a good value.	
Purchase intention (PI)	Please fill out the below questions about purchase intention in MEC during the coronavirus pandemic.	Venkatesh et al. (43)
	PI1. I intend to purchase drugs online.	Kucukusta et al. (44)
	PI2. I predict I would purchase drugs from MEC platforms.	
	PI3. I plan to purchase drugs from MEC platforms.	
	PI4. I will strongly recommend others purchase drugs from MEC platforms.	

All the items were adapted from earlier research but tailored to meet the context of this study. The variables were measured using the following sources: ATT from Taylor and Todd (40); SN from Venkatesh and Davis (41) and Mathieson (42); PBC from Taylor and Todd (40); PV from Venkatesh et al. (37); PI from Venkatesh et al. (43) and Kucukusta et al. (44). The scale ranged from 1 (strongly disagree) to 7 (strongly agree) on a seven point Likert scale. Prior to data collection, the survey was pre-tested and piloted among a selection of participants to gauge their comprehension of the problems it raised and to enhance the caliber of the research findings. Based on responses from the participants, the questionnaire was modified for further data collection.

### 3.2. Data collection

We conducted an online anonymous cross-sectional survey in July 2022 in China. The reason for choosing China as our survey location is because the prosperity of e-commerce offers enormous potential for MEC (17). The questionnaire was designed through Credamo, a data consulting company providing large-scale research, data collection, and business application solutions for research institutions and enterprises. The questionnaire was written in Chinese to ensure that each question item was fully understood. The data were gathered through Credamo. A sample of 489 responses was obtained. We excluded invalid surveys such as those with incorrectly answered screening questions or those where the respondents had never used MEC platforms before. Eventually, 414 valid surveys were used for the final sample.

### 3.3. Demographic statistics

Table 2 shows the demographics of the respondents. The percentages of male and female respondents are 44.4 and 55.6%. The most common monthly income in our sample is under 5,001 RMB (47.3%). A total of 192 (46.4%) participants come from East China, and 222 (53.6%) participants come from Midwest China.

**Table 2:** Demographic of respondents.

**Items**	**Category**	**Frequency**	**Percentage (%)**
Gender	Male	184	44.4
	Female	230	55.6
Monthly income (yuan) (1 yuan = 0.105 USD^*^)	Less than 5,001	196	47.3
	5,001–8,000	100	24.2
	8,001–15,000	94	22.7
	15,001–20,000	16	3.9
	20,001 above	8	1.9
Region	East	192	46.4
	Midwest	222	53.6

^*^As of October 5, 2022.

## 4. Results

### 4.1. Measurement model assessment

To examine the variables' reliability, Cronbach's alpha coefficient of each construct is recommended to be above 0.7 (45). The results showed that Cronbach's alpha coefficient of each construct above 0.7 (Table 3).

**Table 3:** Reliability and validity analysis.

**Construct**	**Item**	**Average variance extracted (AVE)**	**Composite reliability (CR)**	**Cronbach's alpha**	**Loadings**
ATT	ATT1	0.608	0.861	0.856	0.831
	ATT2				0.781
	ATT3				0.785
	ATT4				0.717
SN	SN1	0.508	0.755	0.751	0.672
	SN2				0.790
	SN3				0.670
PBC	PBC1	0.527	0.769	0.766	0.792
	PBC2				0.668
	PBC3				0.713
PI	PI1	0.631	0.872	0.869	0.783
	PI2				0.753
	PI3				0.867
	PI4				0.770
PV	PV1	0.582	0.806	0.805	0.692
	PV2				0.773
	PV3				0.818

Model fit indices: $\frac{\chi^{2}}{df}=$ 2.13, GFI = 0.94, AGFI = 0.91, RMSEA = 0.05, PNFI = 0.74, PGFI = 0.67, CFI = 0.96, NFI = 0.93, IFI = 0.96.

Confirmatory factor analysis was carried out to inspect the model fit, validity and composite reliability (CR) using AMOS 24.0. The fitness indices ($\frac{\chi^{2}}{df}$ < 3, GFI > 0.9, AGFI > 0.8, RMSEA < 0.08, PNFI > 0.5, PGFI > 0.5, CFI > 0.9, NFI > 0.9, IFI > 0.9) were introduced to inspect the model fit (46). Table 3 shows that all fit indices exceeded the recommended value.

Furthermore, CR, factor loading, and average variance extracted (AVE) were adopted to test the measurement model's validity. The results showed that the CR values of all latent variables above 0.7, ranging from 0.755 to 0.872 (47). The standardized factor loadings for all items were larger than 0.5 (47). Table 3 shows that the AVE values range from 0.508 to 0.631 and exceed the recommended value (47). Furthermore, the square root of the AVE was compared against the inter-correlations among constructs. Table 4 shows that the diagonal arithmetic square root of AVE is larger than its correlations with the other constructs (45), suggesting that the model has good discrimination validity.

**Table 4:** Distinguishing validity test of model.

**Items**	**ATT**	**SN**	**PBC**	**BI**	**PV**
ATT	**0.780**				
SN	0.271	**0.713**			
PBC	0.356	0.340	**0.726**		
PI	0.668	0.501	0.523	**0.794**	
PV	0.355	0.218	0.274	0.502	**0.763**

Diagonal values are square root of the AVE value (bold values).

### 4.2. Structural model assessment

**Figure 2:**
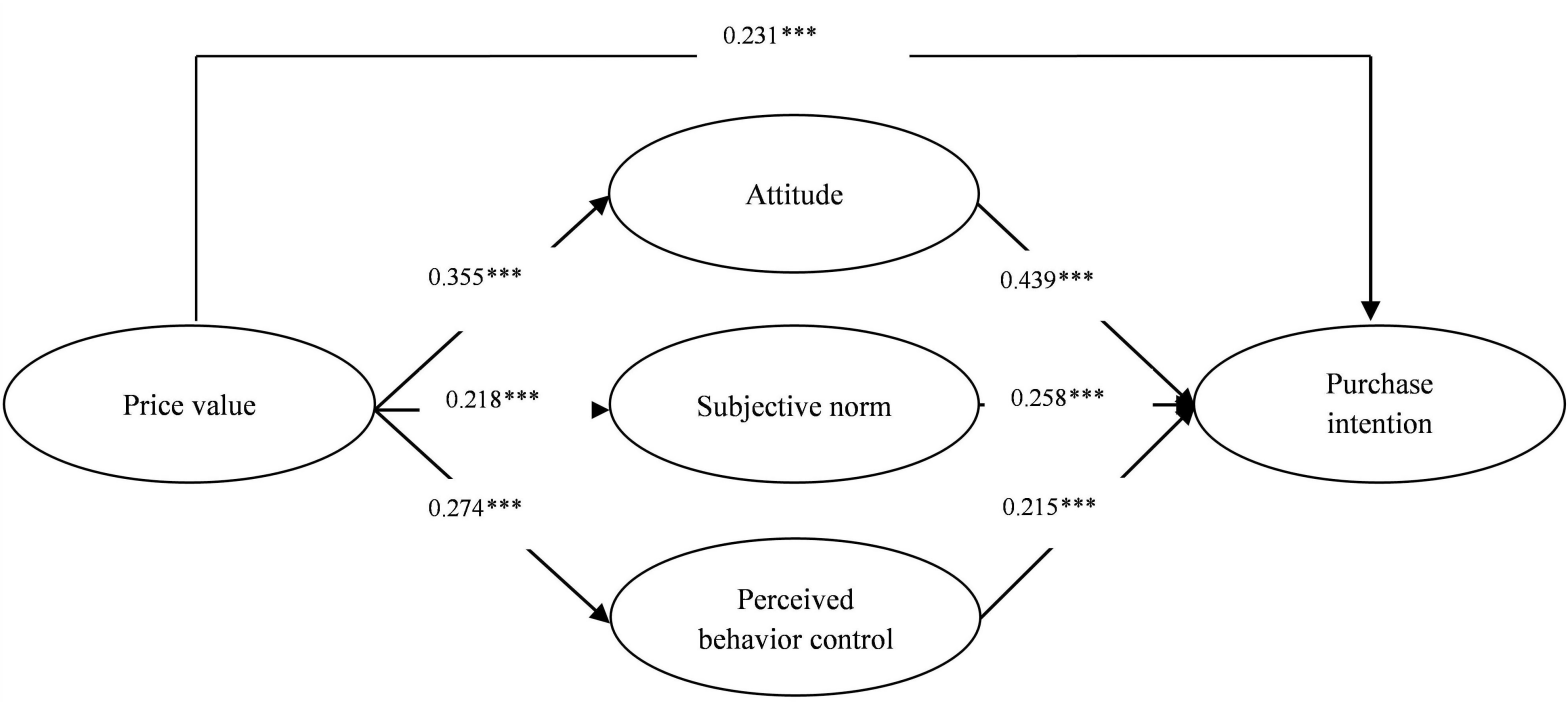
Analysis results of structural model. ***p < 0.001; Model fit indices: χ^2^/*df* = 2.13, GFI = 0.94, AGFI = 0.91, RMSEA = 0.05; PNFI = 0.75, PGFI = 0.67, CFI = 0.96, NFI = 0.93, IFI = 0.96; *R*^2^ for attitude = 0.14, *R*^2^ for subjective norm = 0.07, *R*^2^ for perceived behavior control = 0.10, *R*^2^ for purchase intention = 0.62.

Structural equation modeling (SEM) was used to test the hypotheses in AMOS. Table 5 shows the results and Figure 2 presents the result of SEM graphically depicted. The model fit indices of the structural model ($\frac{\chi^{2}}{df}$ = 2.13, GFI = 0.94, AGFI = 0.91, RMSEA = 0.05, PNFI = 0.75, PGFI = 0.67, CFI = 0.96, NFI = 0.93, IFI = 0.96) all passed the minimum cutoff points (46). ATT (β = 0.439, *p* < 0.001), SN (β = 0.258, *p* < 0.001) and PBC (β = 0.215, *p* < 0.001) were significant predictors of the PI of MEC use during COVID-19. Hence, H1, H2, and H3 are accepted. Moreover, PV significantly affected ATT (β = 0.355, *p* < 0.001), SN (β = 0.218, *p* < 0.001) and PBC (β = 0.274, *p* < 0.001). Accordingly, H4a, H4b, and H4c are accepted. In addition, PV positively affected PI (β = 0.231, *p* < 0.001). Therefore, H4d is accepted.

**Table 5:** Summary of hypotheses test.

**Hypotheses**	**Path**	**Ustd**.	**S.E**.	***T*-value**	***P***	**β**	**Supported**
H1	ATT → PI	0.468	0.058	8.094	***	0.439	Yes
H2	SN → PI	0.263	0.051	5.162	***	0.258	Yes
H3	PBC → PI	0.183	0.043	4.252	***	0.215	Yes
H4a	PV → ATT	0.295	0.052	5.685	***	0.355	Yes
H4b	PV → SN	0.190	0.056	3.387	***	0.218	Yes
H4c	PV → PBC	0.287	0.066	4.360	***	0.274	Yes
H4d	PV → PI	0.205	0.044	4.667	***	0.231	Yes

^***^p < 0.001.

### 4.3. Moderating effects

We conducted a multi-group analysis (MGA) to test the moderating effects of gender (i.e., male and female), income (i.e., high income and low income), and region (i.e., East and Midwest). Differences between gender, income, and regional groups were determined by pairwise comparisons of each relationship between the unconstrained model and constrained model. Here, we impose an equal constraint on each path in the constrained model. A moderating effect exists if the change of chi-square values in the two models is significant (48).

For the gender group comparison (Table 6), gender significantly moderated the relationship between PV and ATT (χ^2^ = 8.920, *p* < 0.05), the moderating effect of PV on PBC was also significant (χ^2^ = 5.273, *p* < 0.05). Compared by males (β = 0.267, *p* < 0.01), ATT of females (β = 0.434, *p* < 0.001) was more likely be influenced by PV. Furthermore, PV had a significant impact on PBC among females (β = 0.404, *p* < 0.001), but the effect of PV on PBC for male group was not significant (β = 0.160, n.s.). Overall, the model explained 58.3% of the variance in PI in the male group and 64.6% variance in the female group.

**Table 6:** Path coefficients comparison of MGA by gender.

**Hypothesis**	**Path**	**Gender**				**Path difference**
		**Male (*****n*** = **184)**		**Female (*****n*** = **230)**		
		β	***t*** **-statistic**	β	***t*** **-statistic**	**Δ**χ^2^ **(*****df*** = **1)**
sH1	ATT → PI	0.418	4.547^***^	0.503	6.826^***^	0.245
H2	SN → PI	0.210	2.519^*^	0.343	5.241^***^	1.581
H3	PBC → PI	0.342	4.085^***^	0.215	3.200^**^	0.439
H4a	PV → ATT	0.267	2.723^**^	0.434	4.991^***^	8.920^**^
H4b	PV → SN	0.189	1.916	0.273	3.131^**^	1.202
H4c	PV → PBC	0.160	1.719	0.404	4.530^***^	5.273^*^
H4d	PV → PI	0.309	4.000^***^	0.143	1.918	0.737
Explained variance in the overall model		58.3%		64.6%		

^*^p < 0.05; ^**^p < 0.01; ^***^p < 0.001.

Table 7 shows the income group comparison. The effect of PV on ATT was significantly different between high- and low-income patients (χ^2^ = 6.043, *p* < 0.05). PV had a stronger effect on ATT for low-income patients (β = 0.420, *p* < 0.001) than for high-income patients (β = 0.268, *p* < 0.05). Overall, the model explained 61.5% of the variance in PI for the high-income group and 62.2% for the low-income.

**Table 7:** Path coefficients comparison of MGA by income.

**Hypothesis**	**Path**	**Income**				**Path difference**
		**High (**≥**5,001) (*****n*** = **218)**		**Low (**<**5,001) (*****n*** = **196)**		
		β	***t*** **-statistic**	β	***t*** **-statistic**	**Δ**χ^2^ **(*****df*** = **1)**
H1	ATT → PI	0.514	5.594^***^	0.432	5.721^***^	1.361
H2	SN → PI	0.301	3.145^**^	0.300	4.416^***^	0.03
H3	PBC → PI	0.176	2.059^*^	0.294	3.982^***^	1.566
H4a	PV → ATT	0.268	3.009^*^	0.420	4.578^***^	6.043^*^
H4b	PV → SN	0.318	3.387^***^	0.179	1.947	0.252
H4c	PV → PBC	0.220	2.558^*^	0.329	3.491^***^	2.027
H4d	PV → PI	0.191	2.583^*^	0.211	2.689^**^	0.71
Explained variance in the overall model		61.5%		62.2%		

^*^p < 0.05; ^**^p < 0.01; ^***^p < 0.001.

Table 8 presents the region group comparison. It was found that PV had a stronger effect on the ATT for patients from Midwest China (β = 0.463, *p* < 0.001) than East China (β = 0.193, *p* < 0.05), and the moderating effects was significant (χ^2^ = 7.564, *p* < 0.001). The region also significantly moderated the relationship between PBC and PI (χ^2^ = 13.866, *p* < 0.001). PBC significantly affected the PI for patients from Midwest China (β = 0.411, *p* < 0.001), but the effect was not significant in patients from East China (β = 0.078, n.s.). The moderating effect of PV on PBC was significant between patients from East and Midwest China (χ^2^ = 3.953, *p* < 0.05). PV was a significant determinant of PBC for patients from Midwest China (β = 0.404, *p* < 0.001), but not for patients from East China (β = 0.149, n.s.). Overall, the model explained 69.5% of the variance in PI of the East China group and 63.0% of the Midwest China group.

**Table 8:** Path coefficients comparison of MGA by region.

**Hypothesis**	**Path**	**Region**				**Path difference**
		**East (*****n*** = **192)**		**Midwest (*****n*** = **222)**		
		β	***t*** **-statistic**	β	***t*** **-statistic**	**Δ**χ^2^ **(*****df*** = **1)**
H1	ATT → PI	0.561	6.372^***^	0.420	5.618^***^	2.203
H2	SN → PI	0.338	4.27^***^	0.266	4.031^***^	0.006
H3	PBC → PI	0.078	1.176	0.411	5.289^***^	13.866^***^
H4a	PV → ATT	0.193	2.119^*^	0.463	5.292^***^	7.564^***^
H4b	PV → SN	0.353	3.656^***^	0.199	2.308^*^	1.555
H4c	PV → PBC	0.149	1.676	0.404	4.457^***^	3.953^*^
H4d	PV → PI	0.296	3.881^***^	0.118	1.552	1.719
Explained variance in the overall model		69.5%		63%		

^*^p < 0.05; ^***^p < 0.001.

## 5. Discussion

This research aims to search for the mechanism of chronic patients' PI in MEC during the COVID-19 pandemic. PV is added to the model based on the TPB framework to explore the mechanism of chronic patients' PI in MEC. The multi-group analysis is conducted by gender, income, and region. The results verify the importance of ATT, SN, PBC, and PV on chronic patients' PI in MEC. The results supported all seven hypotheses and revealed the gender, income, and region differences in the structural relationships among the variables.

First, ATT delivers the most positive effect on PI in MEC, consistent with earlier research on online shopping consumer behavior (49–51). The COVID-19 pandemic has sparked a sharp growth in retail digitalization. Wang et al. (52) found that shoppers' ATT was the strongest contributor to their online PI during the coronavirus pandemic. Patients' PI is often affected by their ATT. On the one hand, purchasing drugs online provides patients with utilities such as instant feedback and fast and reliable home delivery services (17). On the other hand, e-commerce industry has stepped to the maturity stage in China. Purchasing drugs from MEC platforms is also acceptable when patients can get the same quality and service as offline pharmacies. The more patients know about the dominance of MEC, the stronger their ATT to purchasing drugs online; as a result, the PI in MEC will become stronger. Moreover, SN has a relatively weak effect on patients' PI in MEC, which is consistent with the earlier research on exploring online shopping consumer behavior using the TPB model (53, 54). With the perceptions of online shopping, consumers' online PI can also be affected by SN during the COVID-19 pandemic (54). Therefore, patients' PI in MEC is recognized, encouraged, and implemented by SN. Notably, PBC in MEC has a positive but less significant effect than ATT and SN on PI, meaning that patients have sufficient knowledge of different aspects of purchasing drugs online, thus prompting them to adopt MEC platforms. This finding is coherent with former research on consumers' online purchase behavior (55, 56). The reason for such a relationship may be due to the simple and legible online shopping procedure of the MEC platforms (57). Almost all mainstream Chinese MEC platforms provide buyers with a simple and legible shopping process. Therefore, patients' PBC has little influence on PI in MEC.

Second, PV has significant influence on ATT, SN, PBC and PI; PV has the strongest positive effect on ATT, which is consistent with the earlier research. Cheah et al. (58) found that fair-priced and good-value products positively affected consumers' ATT when they used electronic deals. Value perception has a significant correlation with consumers' PI (59). When the benefits overweight the costs, customers perceived the valuation of the product or service (60). The more the expected reduction in long-term costs, the greater the PV perceived (61). Furthermore, PV positively affects patients' ATT in MEC. When patients understand that they can benefit from online drugs purchasing, they will show positive ATT toward purchasing drugs online during the COVID-19 pandemic. Additionally, PV can affect PI through the mediating effect of ATT. The empirical results also demonstrate that PV can influence PI *via* the mediating of SN and PBC. PV plays a vital role in guiding patients' PI in MEC. In China, MEC platforms provide a wide variety of medicines and health products with rapid delivery and excellent services. Owing to enhanced ties among parties related to the industry and digital tools, online sales of prescription drugs have commenced and launched nationwide under the Chinese government administration. In some areas of China, patients can purchase drugs online using medical insurance, which significantly influences of chronic patients' PI.

Finally, this study used MGA to explore the moderating effects of gender, income, and region. Gender is a significant moderating variable from PV to ATT. Compared to males, females' ATT in MEC can be stronger affected by PV. This finding is coherent with former research. Hou et al. (62) identified that females were more likely than males to be bargain hunters in online auctions. Females had significantly higher price consciousness than males (63). As a result, PV seems to exert a greater influence on female patients than on male patients. Regarding income, the effect of PV on ATT is stronger for low-income patients than for high-income patients. It means that if PV is beyond willingness-to-pay levels, the intention to purchase drugs online of low-income patients is more affected by PV than high-income patients in the COVID-19 era. The result is coherent with former research on customer online behavior during the COVID-19 pandemic (64). It is also consistent with the consumption theory. During the COVID-19 pandemic, people with lesser incomes could have tighter budgets and more pressing financial needs, thereby necessitating government relief plans. Furthermore, the region was also a significant moderating variable from PV to ATT. The ATT in MEC of patients from Midwest China may be more affected by PV than that of patients from East China because of the regional development distinction.

## 6. Conclusions

The results of this study make several theoretical contributions. First, this study explores the influencing mechanism of chronic patients' PI in MEC during the COVID-19 pandemic in China which was not discussed by previous research. Thus the results are supposed to contribute to promoting the adoption of MEC among patients with chronic diseases. Second, this study extended the TPB model by integrating the PV variable into the original TPB framework to complement the understanding of patients' PI in MEC. Finally, while examining the adoption of MEC, subgroups categorized by demographic of respondents exhibit different patterns, which may shed light on how to impulse patients' PI in MEC based on the characteristics of different consumer subgroups. The findings of this study are as follows: (i) ATT, SN, and PBC had a significant influence on patients' PI in MEC, and ATT had the strongest effect on the PI. (ii) PV had a significant effect on ATT, SN, PBC, and PI, with PV having the strongest effect on ATT. (iii) In the aspect of mediating effect, PV had a significant indirect influence on PI in MEC through ATT, SN, and PBC. (iv) Gender, income, and region can significantly moderate the relationship between PV and ATT.

Based on the above-mentioned findings, the practical contributions of this study are several: First, as ATT is an important driving factor of MEC adoption, it is important for online drug retailers, MEC platforms, and the government to improve patients' ATT in MEC. With the permission of the government, MEC platforms can launch the “medicine + doctor” model, and provide clinical service and medication guidance to establish and enhance positive ATT in MEC among patients with chronic diseases. Furthermore, MEC platforms can adopt the online-to-offline model to provide services to patients. Patients can order drugs online and pick up drugs in offline pharmacies where additional medication guidance can provide for them. Second, according to our findings, PV has a significant effect on ATT, SN, PBC, and PI. Therefore, it is necessary to strengthen the perception of PV in connection to MEC during the pandemic prevention and control period. For instance, as medical insurance in China is regulated by areas, policymakers should put posit integrate medical insurance and ensure that patients can pay for online drugs purchasing by medical insurance, which may greatly improve patients' ATT and PI in MEC. In the case where medical insurance has not been fully launched, MEC platforms and medical enterprises can conduct chronic care plans, and medical enterprises can provide subsidies to reduce the burden of medication for patients with chronic diseases. Third, since patients' traits, such as gender, income, and region can moderate the impact of some antecedents, targeted strategies can be developed to improve the PI of different chronic patients groups. For instance, patients from Midwest China should receive extra attention, as their ATTs in MEC tend to be more affected by PV than patients from East China. Marketers should also provide corresponding marketing strategies for male and female subgroups, or high- and low-income patients, as the effect of PV on these subgroups' ATTs in MEC are significantly different.

This is the first study to investigate the purchase intention among patients with chronic diseases in MEC during the COVID-19 pandemic. For patients, MEC can provide a way for them to purchase drugs online and reduce the risk of infection. For the government, MEC can relieve the burden on medical resources and decrease community transmission. For MEC platforms, online drug retailers, and medical enterprises, MEC makes it possible for them to sell drugs online under strict laws and regulations, and excellent services will make them even more competitive. The results of the study explored the factors that affect patients' purchase intention in MEC. For the government, forwarding the integration of medical insurance and making sure patients can use medical insurance to purchase drugs online will significantly influence their purchase intention in MEC. For MEC platforms, online drug retailers, and medical enterprises, providing convenience, excellent and personalization service for patients can greatly improve their ATT in MEC. Under the joint efforts of government and enterprises, MEC will develop healthily and serve public health.

The limitations of our study should be acknowledged. First, the questionnaire survey was geographically limited. Future studies should explore how social and cultural differences affect patients' online PI. Second, this study extended the TPB model by introducing just a single variable, PV. Future research could add additional variables within the same context to increase the explained variance.

## Data availability statement

The raw data supporting the conclusions of this article will be made available by the authors, without undue reservation.

## Ethics statement

Ethical review and approval was not required for the study on human participants in accordance with the local legislation and institutional requirements. Written informed consent from the participants was not required to participate in this study in accordance with the national legislation and the institutional requirements.

## Author contributions

LH and XH participated in designing their study, data analysis, and the manuscript, involved in the investigation, and the supervision. All authors contributed to this work and approved the manuscript.

## References

[1] World Health Organization. WHO Director-General's Opening Remarks at the Media Briefing on COVID-19. Geneva: World Health Organization (2020). Available online at: https://www.who.int/director-general/speeches/detail/who-director-general-s-opening-remarks-at-the-media-briefing-on-covid-19-−11-march-2020 (accessed March 11, 2020).

[2] Marin-Garcia D Moyano-Campos JJ Bienvenido-Huertas JD. Distances of transmission risk of COVID-19 inside dwellings and evaluation of the effectiveness of reciprocal proximity warning sounds. Indoor Air. (2020) 31:335–47. 10.1111/ina.12738 32866286

[3] Conly J Seto WH Pittet D Holmes A Chu M Hunter PR. Use of medical face masks vs. particulate respirators as a component of personal protective equipment for health care workers in the context of the COVID-19 pandemic. Antimicrob Resist Int. (2020) 9:126. 10.1186/s13756-020-00810-w 32762735 PMC7406874

[4] Chinese Center for Disease Control Prevention. COVID-19 Disease Prevention and Control Guideline. Beijing: Chinese Center for Disease Control and Prevention (2022). Available online at: https://www.chinacdc.cn/jkzt/crb/zl/szkb_11803/jszl_11815/202206/t20220628_259882.html (accessed June 28, 2022).

[5] Zhao H Liu ZX Li M Liang LJ. Optimal monitoring policies for chronic diseases under healthcare warranty. Sociol Econ Plan Sci. (2022) 84:101384. 10.1016/j.seps.2022.10138434442225

[6] Currie K Price L Curran E Bunyan D Knussen C. Acceptability of temporary suspension of visiting during norovirus outbreaks: investigating patient, visitor and public opinion. J Hosp Infect. (2016) 93:121–6. 10.1016/j.jhin.2015.12.011 26874935 PMC4898206

[7] Li SR Liu Y Su J Luo X Yang X. Can e-commerce platforms build the resilience of brick and mortar businesses to the COVID-19 shock? Electron Commer Res. (2022) 22:1–31. 10.1007/s10660-022-09563-7

[8] Nguyen J Le QV Ha JT. Impacts of health and safety concerns on e-commerce and service reconfiguration during the COVID-19 pandemic: insights from an emerging economy. Serv Sci. (2021) 13:227–42. 10.1287/serv.2021.0279

[9] Orji IJ Ojadi F Okwara UK. The nexus between e-commerce adoption in a health pandemic and firm performance: the role of pandemic response strategies. J Bus Res. (2022) 145:616–35. 10.1016/j.jbusres.2022.03.034

[10] Yang HZ Peng ZY Guo XT Lai KH. Balancing online pharmacy services for patient adherence: a stimulus-organism-response perspective. Internet Res. (2021) 31:2000–32. 10.1108/INTR-10-2020-0603

[11] EqualOcean Intelligence,. China MEC Development White Paper in 2020. New York, NY: EqualOcean Intelligence (2020). Available online at: https://www.iyiou.com/research/20200506709 (accessed May 06, 2020).

[12] World Health Organization. Report of the WHO-China Joint Mission on Coronavirus Disease 2019 (COVID-19). Geneva: World Health Organization (2020). Available online at: https://www.who.int/publications/i/item/report-of-the-who-china-joint-mission-on-coronavirus-disease-2019-(covid-19) (accessed February 28, 2020).

[13] The Central People's Government of the People's Republic of China. Guiding Opinions on Promoting 'Internet +' Medical Insurance Services During the Prevention and Control of Novel Coronavirus Pneumonia. Beijing: The Central People's Government of the People's Republic of China (2020). Available online at: http://www.gov.cn/zhengce/zhengceku/2020-03/03/content_5486256.htm (accessed February 28, 2020).

[14] Liu X Xu YC Yang X. Disease profiling in pharmaceutical E-commerce. Exp Syst Appl. (2021) 178:115015. 10.1016/j.eswa.2021.115015

[15] Sreejesh S Sarkar JG Sarkar A. Digital healthcare retail: role of presence in creating patients' experience. Int J Retail Distrib. (2022) 50:36–54. 10.1108/IJRDM-12-2020-0514

[16] Zehnder S Bruppacher R Ruppanner H Hersberger KE. Swiss community pharmacies' on the Web and pharmacists' experiences with E-commerce: longitudinal study and Internet-based questionnaire survey. J Med Internet Res. (2004) 6:e9. 10.2196/jmir.6.1.e9 15111275 PMC1550588

[17] Ma L. Understanding non-adopters' intention to use internet pharmacy: revisiting the roles of trustworthiness, perceived risk and consumer traits. J Eng Technol Manag. (2021) 59:101613. 10.1016/j.jengtecman.2021.101613

[18] Bosquez NGC Arias-Bolzmann LG Quiroz AKM. The influence of price and availability on university millennials' organic food product purchase intention. Br Food J. (2022) 125:1340. 10.1108/BFJ-12-2021-1340

[19] Al-Debei MM Al-Lozi E Papazafeiropoulou A. Why people keep coming back to Facebook: explaining and predicting continuance participation from an extended theory of planned behaviour perspective. Decis Support Syst. (2013) 55:43–54. 10.1016/j.dss.2012.12.032

[20] Liu YP Shi HK Li YC Amin A. Factors influencing Chinese residents' post pandemic outbound travel intentions: an extended theory of planned behavior model based on the perception of COVID-19. Tour Rev. (2020) 76:871–91. 10.1108/TR-09-2020-0458

[21] Ang T Wei SQ Arli D. Social distancing behavior during COVID-19: a TPB perspective. Mark Intell Plan. (2021) 39:809–24. 10.1108/MIP-08-2020-0352

[22] Ajzen I Timko C. Correspondence between health attitudes and behavior. Basic Appl Soc Psych. (1986) 7:259–76. 10.1207/s15324834basp0704_2

[23] Nguyen MN Potvin L Otis J. Regular exercise in 30- to 60-year-old men: combining the stages-of-change model and the theory of planned behavior to identify determinants for targeting heart health interventions. J Commun Health. (1997) 22:233–46. 10.1023/a:1025196218566 9247847

[24] Ajzen I. The theory of planned behavior. Organ Behav Hum Decis Process. (1991) 50:179–211. 10.1016/0749-5978(91)90020-T

[25] Crawford SY. Internet pharmacy: issues of access, quality, costs, and regulation. J Med Syst. (2003) 27:57–65. 10.1023/A:1021009212905 12617198

[26] Brown J Li CH. Characteristics of online pharmacy users in a nationally representative sample. J Am Pharm Assoc. (2014) 54:289–94. 10.1331/JAPhA.2014.13169 24816356

[27] Fittler A Vida RG Káplár M Botz L. Consumers turning to the internet pharmacy market: cross-sectional study on the frequency and attitudes of hungarian patients purchasing medications online. J Med Internet Res. (2018) 20:e11115. 10.2196/11115 30135053 PMC6125612

[28] Liu JF Zhou YY Jiang XY Zhang W. Consumers' satisfaction factors mining and sentiment analysis of B2C online pharmacy reviews. BMC Med Inform Decis. (2020) 20:194. 10.1186/s12911-020-01214-x 32807175 PMC7433132

[29] Chang F Liu X. Influencing mechanism of consuming intention in pharmacy among patients with chronic diseases-empirical study from the perspective of the theory of planned behavior. Chin Gen Pract. (2015) 18:1431–5. 10.3969/j.issn.1007-9572.2015.12.015

[30] Ajen I. Attitudes, Personality and Behavior. 2nd ed. Maidenhead: Open University Press (2011).

[31] Ajzen I Albarracin D Hornik R. Prediction and Change of Health Behavior: Applying the Reasoned Action Approach. New York, NY: Psychology Press (2007). 10.4324/9780203937082

[32] Hossain MB Alam MZ Islam MS Sultan S Faysal MM Rima SX. Health belief model, theory of planned behavior, or psychological antecedents: what predicts COVID-19 vaccine hesitancy better among the Bangladeshi adults? Front Public Health. (2021) 9:711066. 10.3389/fpubh.2021.711066 34490193 PMC8418098

[33] Ng TKC Lo MF Fong BYF Yee HHL. Predictors of the intention to use traditional Chinese medicine (TCM) using extended theory of planned behavior: a cross-sectional study among TCM users in Hong Kong. BMC Compl Med. (2022) 22:113. 10.1186/s12906-022-03598-x 35459198 PMC9028891

[34] Weber EU Blais AR Betz NE. A domain-specific risk-attitude scale: measuring risk perceptions and risk behaviors. J Behav Decis Making. (2002) 15:263–90. 10.1002/bdm.414

[35] Ricci EC Banterle A Stranieri S. Trust to go green: an exploration of consumer intentions for eco-friendly convenience food. Ecol Econ. (2018) 148:54–65. 10.1016/j.ecolecon.2018.02.010

[36] Ajzen I. Perceived behavioral control, self-efficacy, locus of control, and the theory of planned behavior. J Appl Soc Psychol. (2002) 32:665–83. 10.1111/j.1559-1816.2002.tb00236.x

[37] Venkatesh V Thong JYL Xu X. Consumer acceptance and use of information technology: extending the unified theory of acceptance and use of technology. MIS Quart. (2012) 36:157–78. 10.2307/41410412

[38] Jiang PJ Balasubramanian SK Lambert ZV. Consumers' value perceptions of e-customization: a model incorporating information framing and product type. J Consum Mark. (2014) 31:54–67. 10.1108/JCM-04-2013-0534

[39] Zeithaml VA. Consumer perceptions of price, quality, and value: a means-end model and synthesis of evidence. J Mark. (1988) 52:2–22. 10.1177/002224298805200302

[40] Taylor S Todd PA. Understanding information technology usage: a test of competing models. Inform Syst Res. (1995) 6:144–76. 10.1287/isre.6.2.144

[41] Venkatesh V Davis FD. A theoretical extension of the technology acceptance model: four longitudinal field studies. Manag Sci. (2000) 46:186–204. 10.1287/mnsc.46.2.186.11926

[42] Mathieson K. Predicting user intentions: comparing the technology acceptance model with the theory of planned behavior. Inform Syst Res. (1991) 2:173–91. 10.1287/isre.2.3.173

[43] Venkatesh V Morris MG Davis GB Davis FD. User acceptance of information technology: toward a unified view. MIS Quart. (2003) 27:425–78. 10.2307/30036540

[44] Kucukusta D Law R Besbes A Legohérel P. Re-examining perceived usefulness and ease of use in online booking: the case of Hong Kong online users. Int J Contemp Hosp Manag. (2015) 27:185–98. 10.1108/IJCHM-09-2013-0413

[45] Hair JF Anderson RE Babin BJ Black WC. Multivariate Data Analysis: A Global Perspective. London: Pearson (2010).

[46] Cui Y Mou J Cohen J Liu YP. Understanding information system success model and valence framework in sellers' acceptance of cross-border e-commerce: a sequential multi-method approach. Electron Commer Res. (2019) 19:885–914. 10.1007/s10660-019-09331-0

[47] Hair J Hollingsworth CL Randolph AB Chong AYL. An updated and expanded assessment of PLS-SEM in information systems research. Ind Manag Data Syst. (2017) 17:442–58. 10.1108/IMDS-04-2016-0130

[48] Byrne BM. Structural Equation Modeling With AMOS: Basic Concepts, Applications, and Programming. New York, NY: Taylor & Francis Group (2010).

[49] Hansen T Jensen JM Solgaard HS. Predicting online grocery buying intention: a comparison of the theory of reasoned action and the theory of planned behavior. Int J Inform Manag. (2004) 24:539–50. 10.1016/j.ijinfomgt.2004.08.004

[50] Piroth P Ritter MS Rueger-Muck E. Online grocery shopping adoption: do personality traits matter? Br Food J. (2020) 122:957–75. 10.1108/BFJ-08-2019-0631

[51] Kao WK L'Huillier EA. The moderating role of social distancing in mobile commerce adoption. Elect Commer R A. (2022) 52:101116. 10.1016/j.elerap.2021.101116 35013678 PMC8734084

[52] Wang XQ Wong YD Chen TY Yuen KF. An investigation of technology-dependent shopping in the pandemic era: integrating response efficacy and identity expressiveness into theory of planned behavior. J Bus Res. (2022) 142:1053–67. 10.1016/j.jbusres.2022.01.042

[53] Tang HL Rasool Z Khan MA Khan AI Khan F Ali H. Factors affecting e-shopping behaviour: application of theory of planned behaviour. Behav Neurol. (2021) 2021:1664377. 10.1155/2021/1664377 34858540 PMC8632471

[54] Akar E. Customers' online purchase intentions and customer segmentation during the period of COVID-19 pandemic. J Internet Commer. (2021) 20:371–401. 10.1080/15332861.2021.1927435

[55] Adiyoso WW. Social distancing intentions to reduce the spread of COVID-19: the extended theory of planned behavior. BMC Public Health. (2021) 21:1836. 10.1186/s12889-021-11884-5 34635071 PMC8503732

[56] Rehman SU Bhatti A Mohamed R Ayoup H. The moderating role of trust and commitment between consumer purchase intention and online shopping behavior in the context of Pakistan. J Glob Entrep Res. (2019) 9:43. 10.1186/s40497-019-0166-2

[57] Amin M Rezaei S Tavana FS. Gender differences and consumer' repurchase intention: the impact of trust propensity, usefulness, and ease of use for implication of innovative online retail. Int J Innov Learn. (2015) 17:217–33. 10.1504/IJIL.2015.067409

[58] Cheah I Phau I Liang JH. Factors influencing consumers' attitudes and purchase intentions of e-deals. Mark Intell Plan. (2015) 33:763–83. 10.1108/MIP-05-2014-0081

[59] Olsen LL Johnson M. Service equity, satisfaction, and loyalty: from transaction-specific to cumulative evaluations. J Serv Res. (2003) 5:184–95. 10.1177/1094670502238914

[60] Vafaei-Zadeh A Hanifah H Teoh A Wong TK Nawaser K. Modelling electric vehicle purchase intention among generation Y consumers in Malaysia. Res Transp Bus Manag. (2022) 43:100784. 10.1016/j.rtbm.2022.100784

[61] Zhang YX Xiao CC Zhou GH. Willingness to pay a price premium for energy-saving appliances: role of perceived value and energy efficiency labeling. J Clean Prod. (2020) 242:118555. 10.1016/j.jclepro.2019.118555

[62] Hou JW Elliott K. Gender differences in online auctions. Electron Commer R A. (2016) 17:123–33. 10.1016/j.elerap.2016.04.004

[63] Seock YK Bailey LR. The influence of college students' shopping orientations and gender differences on online information searches and purchase behaviours. Int J Consum Stud. (2008) 32:113–21. 10.1111/j.1470-6431.2007.00647.x

[64] Yue W Liu N Zheng QJ Wang HH. Does the COVID-19 pandemic change consumers' food consumption and willingness-to-pay? The case of China Foods. (2021) 10:2156. 10.3390/foods10092156 34574266 PMC8470711

